# Regression discontinuity of blood culture contamination rate after changing of disinfectants: retrospective observational study

**DOI:** 10.1038/s41598-021-00498-x

**Published:** 2021-10-27

**Authors:** Koshi Ota, Daisuke Nishioka, Yuri Ito, Emi Hamada, Naomi Mori, Tomonobu Nishii, Kanna Ota, Yuriko Shibata, Akira Takasu

**Affiliations:** 1 Department of Emergency Medicine, Osaka Medical College, 2-7 Daigaku-machi, Takatsuki City, Osaka 596-8686 Japan; 2 Research and Development Center, Osaka Medical College, Takatsuki City, Japan; 3 Department of Nursing, Osaka Medical College Hospital, Takatsuki City, Japan; 4 Department of Clinical Laboratory, Osaka Medical College Hospital, Takatsuki City, Japan

**Keywords:** Microbiology, Medical research

## Abstract

Blood cultures are indispensable for detecting life-threatening bacteremia. Little is known about associations between contamination rates and topical disinfectants for blood collection in adults. We sought to determine whether a change in topical disinfectants was associated with the rates of contaminated blood cultures in the emergency department of a single institution. This single-center, retrospective observational study of consecutive patients aged 20 years or older was conducted in the emergency department (ED) of a university hospital in Japan between August 1, 2018 and September 30, 2020. Pairs of blood samples were collected for aerobic and anaerobic culture from the patients in the ED. Physicians selected topical disinfectants according to their personal preference before September 1, 2019; alcohol/chlorhexidine gluconate (ACHX) was mandatory thereafter, unless the patient was allergic to alcohol. Regression discontinuity analysis was used to detect the effect of the mandatory usage of ACHX on rates of contaminated blood cultures. We collected 2141 blood culture samples from 1097 patients and found 164 (7.7%) potentially contaminated blood cultures. Among these, 445 (20.8%) were true bacteremia and 1532 (71.6%) were true negatives. Puncture site disinfection was performed with ACHX for 1345 (62.8%) cases and with povidone-iodine (PVI) for 767 (35.8%) cases. The regression discontinuity analysis showed that mandatory ACHX usage was significantly associated with lower rates of contaminated blood cultures by 9.6% (95% confidence interval (CI): 5.0%–14.2%, *P* < 0.001). Rates of contaminated blood cultures were significantly lower when ACHX was used as the topical disinfectant.

## Introduction

Blood culture is an indispensable test for the detection of life-threatening bacteremia, a condition that is associated with high morbidity and mortality. Contaminated blood cultures can result in unnecessary antibiotic use, longer hospitalization, and increased health care costs^1,2^. Several strategies have been recommended to reduce rates of blood culture contamination^3,4^. However, a systematic review has determined that only sampling from separate venipuncture sites and a well-trained phlebotomy team can achieve this rate reduction^5^. Although topical alcohol/chlorhexidine gluconate (ACHX) reduces blood culture contamination more effectively than aqueous povidone-iodine (PVI)^6–9^, most of the relevant studies were conducted in children; to our knowledge, definitive evidence on this topic had not been reported. At our institution, both agents have been routinely applied as topical disinfectants before blood sampling. Our previous study revealed that use of PVI is an independent risk factor for blood culture contamination^10^. Based on that study, we changed our institutional policy, making ACHX mandatory as the topical disinfectant (unless the patient was allergic to alcohol) as of September 1, 2019.

We hypothesized that the mandatory use of ACHX before blood sampling may have changed the rate of blood culture contamination. To date, few studies (to our knowledge) have examined whether the mandatory use of ACHX before blood sampling reduces the rates of blood culture contamination in adult patients. Therefore, we conducted an observational cohort study using regression discontinuity analysis to compare the rates of blood culture contamination before and after the introduction of mandatory ACHX usage in a single emergency department (ED).

## Methods

### Study design

This single-center, retrospective observational study was conducted at the ED of a university hospital in Japan between August 1, 2018, and September 30, 2020. The hospital is an 882-bed university teaching hospital with 8000 adults presenting at the ED annually. Strengthening the Reporting of Observational studies in Epidemiology (STROBE) guidelines were used to design and report the results of this study. The Institutional Review Board at Osaka Medical College approved the study protocol (No. 675(2476)) and waived the need for written, informed consent. All investigations were carried out in accordance with relevant guidelines and regulations.

### Patients

This study included 1097 consecutive patients aged ≥ 20 years from whom blood was sampled in the emergency room (ER). The inclusion criteria were that all patients were aged ≥ 20 years and that at least one pair of blood cultures was collected in the ER. The exclusion criteria were that blood was sampled elsewhere and that age was < 20 years. If one pair of blood samples was collected at our ED and another was collected elsewhere or not collected, then only the pair collected at our ED was analyzed. One or more of the following comorbidities of the patients were recorded: malignancy, diabetes mellitus, hypertension, prior stroke, dementia, chronic renal insufficiency, liver cirrhosis, and coronary artery disease^11–14^.

### Blood cultures

Nurses and other medical staff at our institution were not permitted to collect blood for blood culture. Only physicians, typically first- or second-year interns, were permitted to collect blood samples in the ED.

Blood (14–20 mL) from peripheral veins or arteries was sampled for aerobic and anaerobic cultures (7–10 mL each) in BacT/Alert FA Plus and FN Plus resin bottles (bioMérieux, Inc., Durham, NC, USA). Prior to August 31, 2019, physicians selected the topical disinfectant (from choices such as 1% ACHX, 10% PVI, alcohol, and others available in the ED) according to their personal preferences. As of August 31, 2019, physicians were to use ACHX only, unless the patient was allergic to alcohol. A blood culture was considered contaminated if one or more of the following organisms were identified in one of the two blood cultures: coagulase-negative staphylococci (CoNS), *Propionibacterium acnes*, *Micrococcus* spp., *Corynebacteria* spp., *Bacillus* spp. other than *Bacillus anthracis*, or *Clostridium perfringens*^15–17^. Polymicrobial cultures with a mixture of contaminant and true pathogen also were regarded as contaminated^14^. Viridans streptococci were regarded as contaminants based on the described criteria^15,16^, but these bacteria were excluded as contaminants at our institute. The blood culture was defined as “negative” when bacterial growth was absent or when a bacterium was regarded by the attending microbiologist as having low pathogenicity. The source of infection was identified based on comparison (by chart review) with other cultures such as those from sputum, urine, ascites, and so on, or with other modalities including ultrasound, X-ray, computed tomography (CT), and magnetic resonance imaging (MRI). Some patients had several sources of infection, for example, pneumonia with urinary tract infection. Details were provided in Table 1.

**Table 1:** Characteristics of enrolled patients.

Characteristics of patients	True bacteremia		Contamination		True negative		*P*
	n = 241		n = 141		n = 715		
Mean age, y (SD)	72.9	(12.8)	72.7	(13.3)	68.3	(17.1)	< 0.001
Male sex, n (%)	145	(60.2)	95	(67.4)	422	(59.0)	0.179
**Major comorbidities, n (%)**							
Malignancy	131	(54.4)	65	(46.1)	300	(42.0)	0.004
Diabetes mellitus	77	(32.0)	39	(27.7)	164	(23.0)	0.018
Hypertension	124	(51.5)	79	(56.0)	266	(37.3)	< 0.001
Previous stroke	29	(12.0)	16	(11.4)	64	(9.0)	0.321
Chronic renal insufficiency	51	(21.2)	35	(24.8)	109	(15.2)	0.007
Liver cirrhosis	35	(14.5)	25	(17.7)	63	(8.8)	0.002
Coronary artery diseases	35	(14.5)	37	(26.2)	105	(14.7)	0.002
Dementia	24	(10.0)	18	(12.8)	56	(7.8)	0.141
Quick SOFA, n (%)						0.019	
0	88	(36.5)	56	(39.7)	286	(40.0)	
1	72	(29.9)	45	(31.9)	268	(37.5)	
2	66	(27.4)	32	(22.7)	140	(19.6)	
3	15	(6.2)	8	(5.7)	21	(2.9)	
**Origin of infection, n (%)**							
Central nervous system	3	(1.2)	5	(3.6)	23	(3.2)	0.211*
Pulmonary	34	(14.1)	56	(39.7)	256	(35.8)	< 0.001
Cardiovascular system	13	(5.4)	2	(1.4)	27	(3.8)	0.143*
Abdomen	63	(26.1)	34	(24.1)	152	(21.3)	0.268
Urinary tract	112	(46.5)	20	(14.2)	149	(20.8)	< 0.001
Skin	16	(6.6)	11	(7.8)	43	(6.0)	0.717
Other	15	(6.2)	16	(11.4)	86	(12.0)	0.04

SD, standard deviation; SOFA, sequential organ failure assessment.

*Fisher exact test was performed because of small number of patients in several cells; other groups were analyzed using two-tailed χ^2^ test and one-way analysis of variance (ANOVA).

Origin of infection means the cause of infection, as judged based on medical chart review including other cultures and various diagnostic modalities.

Central nervous system included meningitis, encephalitis, and brain abscess. Pulmonary included pneumonia, bronchitis, pleuritis, and upper respiratory infection. Cardiovascular system included endocarditis and pericarditis. Abdomen included cholangitis, gastroenteritis, cancer of gastrointestinal tract, hepatitis, cholecystitis, appendicitis, and pancreatitis. Urinary tract included pyelonephritis, cystitis, and prostatitis. Skin included decubitus, cellulitis, impetigo, and erysipelas. Other included febrile neutropenia and cases in which the source of infection could not be identified.

### Statistical analysis

Categorical variables were presented as frequencies and percentages (%); continuous variables were shown as means with standard deviation (SD). Data were compared using two-tailed tests, including one-way analyses of variance (ANOVA), χ^2^, and Fisher exact tests, as appropriate. Samples collected from the same patient but at separate admissions were considered as independent events in the above model. Because blood could be sampled from more than one site, we also included, in the category “Other”, blood sampled from arterial catheters as well as from implanted ports. Because we did not have many topical disinfectants to assess, we included PVI, ACHX, and Other types as categories in the analyses. First, we summarized the characteristics of the blood cultures for the 13 months preceding and 12 months following the change in policy. September 2019 was the “threshold” of this study. We compared three groups (patients with bacteremia, those with negative blood cultures, and those with contaminated blood cultures) for the proportion of ACHX usage, male sex, femoral artery or vein for blood sampling, and elderly who were 60 years of age or older at the time of blood collection during the 26-month study interval (Table 3 and Supplemental Table S1). We then conducted the main analysis using the monthly contamination rate as an outcome variable. We implemented local linear regression to estimate the impact of the changing topical disinfectants at the threshold; that is, the analysis employed a regression-discontinuity test. We chose a local linear specification rather than higher-order polynomials to prevent overfitting of the data and misleading impact estimation. We used a triangular kernel function to give weight to observations near the threshold. In addition, we determined the bandwidth around the threshold using the data-driven optimal bandwidth method. To check for robustness, we created a series of alternative models, employing multiple bandwidths and polynomial orders in our regression equations, and compared the resulting estimates.

We did not impute for missing values. Significance for all statistical findings was taken at *P* < 0.05. All analyses were performed with STATA (version 16.1; Stata Corp., College Station, TX, USA).

## Results

### Baseline characteristics

Data from 2141 blood cultures of 1097 patients were analyzed for the greater-than-2-year study interval. A total of 164 (7.7%) blood cultures from 141 (12.9%) patients were identified as potentially harboring contaminants, among which the most common bacterium was *Staphylococcus epidermidis* in 46 (28.1%) cultures, followed by *Staphylococcus hominis* in 17 (10.4%)*.* Four hundred and forty-five (20.8%) cultures from 241 patients (22.0%) showed true bacteremia, in which the most prevalent microorganism was *Escherichia coli* in 94 (21.2%) cultures*.* One thousand five hundred and thirty-two (71.6%) cultures from 715 patients (65.2%) were true negatives. Among the 241 true bacteremia patients, the largest source of the organism was urinary tract infection (UTI), which constituted 112 (46.5%) cases, among which the most prevalent microorganism was *Escherichia coli*. Among the 141 patients in the contamination group and 715 patients in the true negative group, the largest source of the organism was pulmonary origin, representing 56 (39.7%) and 256 (35.8%) of the respective groups. The proportions of infections from the two sources (UTI and pulmonary) differed significantly from each other in each of the three groups (*P* < 0.001) (Table 1). Femoral sites were most likely to be selected for blood sampling in all three groups, and the proportion of femoral site use (75.6%, 124/164 cultures) in the contamination group was significantly higher than those in the other groups (*P* < 0.001, vs. 53.9% (240/445 cultures) in the true bacteremia group and 50.3% (771/1532 cultures) in the true negative group) (Table 2).

**Table 2:** Characteristics of blood cultures.

	True bacteremia		Contamination		True negative		P
	n = 445		n = 164		n = 1532		
**Site, n (%)**							
CV catheter	14	(3.2)	20	(12.2)	22	(1.4)	< 0.001
Venous catheter	29	(6.5)	4	(2.4)	125	(8.2)	0.014*
Other	16	(3.6)	2	(1.2)	43	(2.8)	0.311*
Venous	146	(32.8)	14	(8.5)	571	(37.3)	< 0.001
Femoral	240	(53.9)	124	(75.6)	771	(50.3)	< 0.001
**Antiseptics, n (%)**							
PVI	165	(37.1)	127	(77.4)	467	(30.5)	< 0.001
ACHX	274	(61.6)	35	(21.3)	1036	(67.6)	< 0.001
Other types	6	(1.4)	2	(1.2)	29	(1.9)	< 0.001
One pair	7	(1.6)	5	(3.1)	42	(2.7)	0.357*

ACHX, 1.0% alcohol/chlorhexidine gluconate; CV, central venous; Femoral, femoral artery or vein; Other types, alcohol and benzalkonium; Other, recently inserted arterial catheter and implanted port; PVI, 10% aqueous povidone-iodine; Venous, venipuncture without catheter insertion; Venous catheter, recently inserted venous catheter.

*Fisher exact test was performed because of small number of patients in several cells; other groups were analyzed by two-tailed χ^2^ test.

Blood sampled from recently inserted central venous catheters conferred the greatest risk for contamination when taken as an independent factor.

One pair of blood samples was collected from each of 54 patients (Table 2). Tables 1 and 2 show other baseline characteristics of the three groups of patients.

### Monthly contamination rate of blood cultures

Table 3 showed monthly data for blood cultures across an interval of 7 months, including 3 months before and 3 months after the implementation of the new policy in September 2019. The Supplemental Table S1 provided more detailed monthly data for blood cultures across the entire 26-month study interval. The proportions (in patients with contaminated blood cultures) of elderly (60 years and older), male sex, and using femoral sites for blood sampling, did not differ significantly (*P* = 0.321, *P* = 0.526, and *P* = 0.215, respectively) (Supplemental Table S1). However, ACHX usage differed significantly among the three groups of patients (Supplemental Table S1). Notably, while the monthly rate of contamination was 5.7–20.0% before September 2019, that rate was 0.0–8.2% after September 2019 (Table 3 and Supplemental Table S1).

**Table 3:** Monthly data for blood cultures in emergency department.

	Jun-19		Jul-19		Aug-19		**Sep-19**		Oct-19		Nov-19		Dec-19	
**Disinfectants**														
PVI	41	(49.4)	53	(65.4)	39	(36.8)	**6**	**(6.0)**	2	(2.9)	2	(3.6)	5	(8.2)
ACHX	37	(44.6)	26	(32.1)	60	(56.6)	**94**	**(94.0)**	67	(97.1)	52	(94.5)	56	(91.8)
Other	5	(6.0)	2	(2.5)	7	(6.6)	**0**	**0.0**	0	0.0	1	(1.8)	0	0.0
**Blood culture**														
True bacteremia	22	(26.5)	24	(29.6)	25	(23.6)	**16**	**(16.0)**	13	(18.8)	18	(32.7)	9	(14.8)
Contamination	6	(7.2)	10	(12.3)	11	(10.4)	**0**	**0.0**	0	0.0	1	(1.8)	5	(8.2)
True negative	55	(66.3)	47	(58.0)	70	(66.0)	**84**	**(84.0)**	56	(81.2)	36	(65.5)	47	(77.0)
**Site**														
CV	3	(3.6)	1	(1.2)	2	(1.9)	**3**	**(3.0)**	1	(1.4)	1	(1.8)	1	(1.6)
Femoral	42	(50.6)	35	(43.2)	59	(55.7)	**47**	**(47.0)**	36	(52.2)	30	(54.5)	38	(62.3)
Venous	25	(30.1)	32	(39.5)	29	(27.4)	**38**	**(38.0)**	26	(37.7)	22	(40.0)	14	(23.0)
Male (%)	55	(66.3)	43	(53.1)	68	(64.2)	**59**	**(59.0)**	33	(47.8)	35	(63.6)	26	(42.6)
Age, y (SD)	68.4	(17.2)	70.0	(12.6)	71.0	(13.5)	**69.1**	**(16.3)**	66.5	(19.7)	73.2	(13.2)	68.9	(16.6)

Total number of blood cultures is categorized as three groups, including true bacteremia, contamination, and true negative. Disinfectants categories indicate the proportions of PVI (10% aqueous povidone-iodine), ACHX (1.0% alcohol/chlorhexidine gluconate), and Other disinfectants usage. Site category does not include Other (recently inserted arterial catheter and implanted port) and Venous catheter (recently inserted venous catheter). September 2019 was at the threshold (institution of policy change; bold font.

### Regression discontinuity analysis

**Figure 1:**
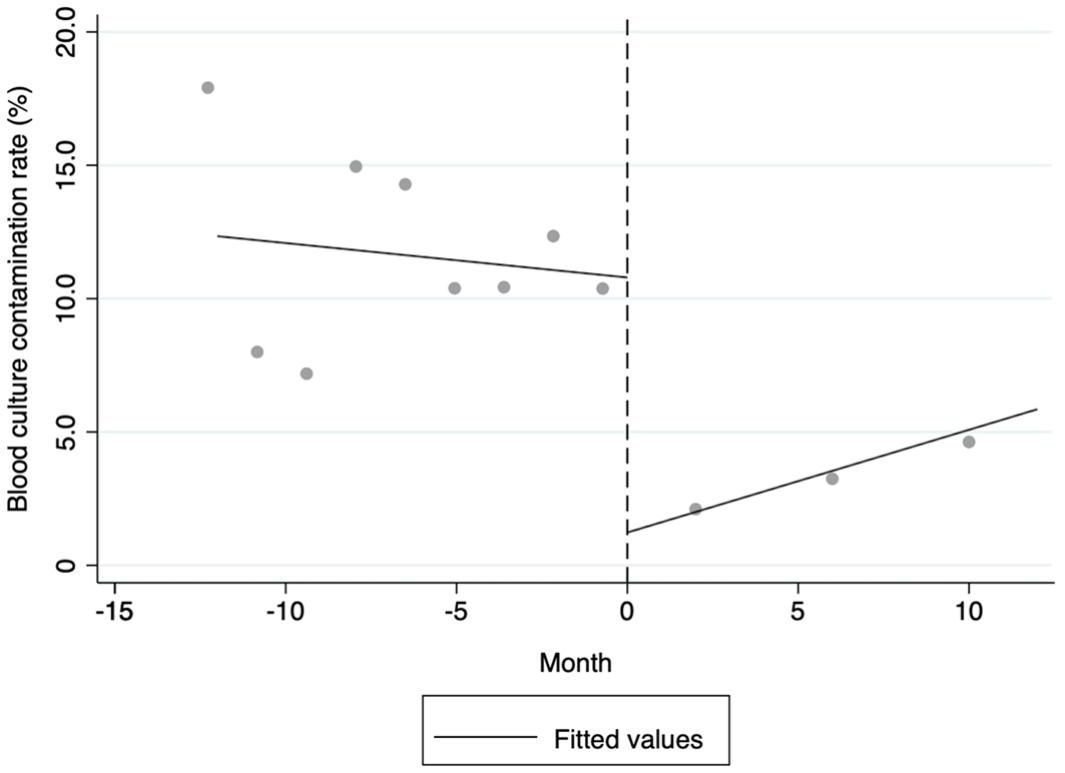
Linear fit of regression discontinuity. Trends in the rates of blood culture contamination from August 2018 to September 2020. The black dashed line indicates the month of September 2019 (the threshold). Gray circles indicate the actual rates of blood culture contamination. The black solid lines indicate the linear fitted values.

The regression discontinuity analysis showed that mandatory ACHX usage was significantly associated with lower rates of contaminated blood cultures (*P* < 0.001). As is shown in Fig. 1, there were significant discontinuities in the rates of blood culture contamination in September 2019. Using this threshold for the blood culture contamination rate, our statistical analysis showed that the intervention of mandatory ACHX usage was associated with a significant lower rate of blood culture contamination, which fell by 9.6% (95% confidence interval (CI): 5.0%–14.2%, *P* < 0.001).

In the sensitivity analysis, mandatory ACHX usage was significantly associated with lower rates of contaminated blood cultures, even when employing different bandwidths and polynomial orders (Table 4).

**Table 4:** Estimates of the effects on contamination of change in topical disinfectants.

Bandwidth for regression discontinuity	Difference (%)	95% confidence interval (%)		
Sep-2018—Sep-2020	− 9.6	− 14.2	–	− 5.0
Nov-2018—Jul-2020	− 8.8	− 13.9	–	− 3.8
Jan-2019—May-2020	− 9.2	− 15.0	–	− 3.4
Mar-2019—Mar-2020	− 10.8	− 17.7	–	− 3.8
May-2019—Jan-2020	− 12.3	− 21.6	–	− 3.0
Jul-2019—Nov-2019	− 10.4	− 16.2	–	− 4.5
**Polynomial order**				
Linear fit	− 9.6	− 14.2	–	− 5.0
Quadratic fit	− 8.1	− 15.5	–	− 0.8
Cubic fit	− 13.2	− 24.6	–	− 1.8
Quartic fit	− 15.4	− 34.3	–	3.5

Regression discontinuity analyses comparing rates of blood culture contamination before versus after September 2019 with different bandwidths.

Polynomial order analyses comparing rates of blood culture contamination before versus after September 2019 with same bandwidth, September 2018 to September 2020.

## Discussion

This single-center, retrospective observational study found that blood samples drawn from sites disinfected with ACHX exhibited a significantly lower proportion of contaminated blood cultures; this change was not apparently associated with a change in blood sampling technique. The most common source of infection among patients with true bacteremia was the urinary tract, and such patients typically presented with pyelonephritis. In contrast, the most prevalent source of infection among patients with contaminated and true negative blood cultures was pulmonary, with most such patients presenting with aspiration pneumonia, as judged by chart review and demonstrated by sputum culture and CT scan.

We also found that femoral sites were likely to be selected; selection of femoral artery for blood collection was significantly associated with contaminated blood cultures.

Several previous reports have described associations between topical disinfectants and blood culture contamination^18,19^. A meta-analysis found that the rate of blood culture contamination is significantly decreased by disinfection with ACHX compared to that seen by disinfection with PVI^6^. However, prior to our facility’s policy change, physicians at our hospital tended to disinfect puncture sites with PVI rather than with ACHX, for the following reasons. Firstly, physicians and ED staff were more familiar with PVI than ACHX because the former has been employed as a skin disinfectant for many years. Secondly, residents and medical students had not been educated about blood culture procedures while at university.

Our previous study revealed that femoral puncture sites comprise an independent risk factor for blood culture contamination^10^. Physicians tend to collect blood from femoral arteries or veins because collection from this site is easier than collection from other sites. Femoral sites have been reported to be colonized more often than other sites^20^, and these colonization events were associated with catheter-related bloodstream infection^21^. However, the proportion of blood draws performed using femoral sites did not differ significantly between patients with and without contaminated blood cultures during the 2-year interval of the present study (Table 3). Thus, our regression discontinuity analysis indicated that the observed change in contamination rate was the result primarily of the change in disinfectants. Furthermore, this regression discontinuity analysis permitted determination of unbiased causal effect estimates, facilitating evaluation of intervention efficacy in a real-world circumstance^22,23^.

Pulmonary infection was the most-frequently found source of infection in patients with contaminated and true negative blood culture. Pulmonary infection may cause pulmonary diseases such as pneumonia, bronchitis, pleuritis, and upper respiratory infection, without resulting in bacteremia. Several studies of patients with pulmonary disease have found that blood cultures provide little diagnostic benefit^24–26^. However, other studies have indicated that blood samples should be cultured from select immunocompromised patients, from individuals with complicated UTIs who are under antibiotic therapy at the time of blood collection, and from patients with suspected endocarditis^25,27^.

Several strategies, including sampling from various venipuncture sites, reliance on a well-trained phlebotomy staff, and the informational intervention and feedback, have been advocated to reduce rates of blood culture contamination^5,28,29^. Topical disinfection with olanexidine, which already is commercially available in Japan, also is expected to reduce the rate of blood culture contamination, given that this disinfectant recently was shown to exhibit stronger bactericidal activity than PVI for surgical site infection^30^.

This study has several limitations. First, our study was observational in design, and some physicians were aware that this study was underway within the ED; hence, these physicians may have been more attentive when collecting blood within the ED than when performing these procedures in the wards. However, this study has continued for more than 2 years; thus, physicians would have become accustomed to being part of this study, which would have reduced potential bias. Second, before the change in policy, physicians were able to select their preferred topical disinfectant for blood sampling; this aspect may have been a confounder, given that multiple studies have shown that contamination is associated more frequently with PVI than with ACHX. However, as shown in Table 3 and the Supplemental Table S1, physicians provided with a choice tended to choose PVI rather than ACHX. Third, this analysis was based on a single-center study, and so might not be generalizable to other hospitals. Nevertheless, our regression discontinuity analysis was robust, leading us to hypothesize that our intervention will be effective in reducing blood culture contamination at other clinical sites.

## Conclusions

This retrospective observational study found that mandatory ACHX usage was significantly associated with lower rates of contaminated blood cultures in adult patients.

### Data availability

The datasets used and/or analyzed during the current study are available from the corresponding author on reasonable request.

## Supplementary Information

Supplementary Table S1.
