# Supplementary material for: Regression discontinuity of blood culture contamination rate after changing of disinfectants: retrospective observational study
Source: Sci Rep. 2021 Oct 27;11:21235. doi: 10.1038/s41598-021-00498-x (PMC8551281; doi:10.1038/s41598-021-00498-x)
Supplement: Supplementary file 1 — Supplementary Table S1. [file 41598_2021_498_MOESM1_ESM.docx]

Supplemental Table S1

Total number of blood cultures is categorized as three groups including true bacteremia, contamination, and true negative. Each category is shown with the proportion of ACHX (1.0% alcohol/chlorhexidine gluconate) usage, male sex, femoral artery or vein for blood sampling, and elderly who were aged 60 years and older.
